# Case report: Biochemical and clinical phenotypes caused by cysteine substitutions in the epidermal growth factor-like domains of fibrillin-1

**DOI:** 10.3389/fgene.2022.928683

**Published:** 2022-08-11

**Authors:** Xin Liu, Kaiqing Liu, Danyao Nie, Jing Zhang, Liyun Zhang, Xinhua Liu, Jiantao Wang

**Affiliations:** ^1^ Shenzhen Eye Hospital, Shenzhen Key Laboratory of Ophthalmology, Affiliated Shenzhen Eye Hospital of Jinan University, Shenzhen, China; ^2^ Guangdong Key Laboratory of Systems Biology and Synthetic Biology for Urogenital Tumors, Shenzhen Second People’s Hospital, The First Affiliated Hospital of Shenzhen University, Shenzhen, China

**Keywords:** Marfan syndrome, FBN1, mutation, EGF-like domain, cysteine

## Abstract

Marfan syndrome, an autosomal dominant disorder of connective tissue, is primarily caused by mutations in the fibrillin-1 (FBN1) gene, which encodes the protein fibrillin-1. The protein is composed of epidermal growth factor-like (EGF-like) domains, transforming growth factor beta-binding protein-like (TB) domains, and hybrid (Hyb) domains and is an important component of elastin-related microfibrils in elastic fiber tissue. In this study, we report a cysteine to tyrosine substitution in two different domains of fibrillin-1, both of which cause Marfan syndrome with ocular abnormalities, in two families. Using protease degradation and liquid chromatography-tandem mass spectrometry analyses, we explored the different effects of substitution of cysteine by tyrosine in an EGF-like and a calcium-binding (cb) EGF-like domain on protein stability. The results showed that cysteine mutations in the EGF domain are more likely to result in altered proteolytic sensitivity and thermostability than those in the cbEGF domain. Furthermore, cysteine mutations can lead to new enzymatic sites exposure or hidden canonical cleavage sites. These results indicate the differential clinical phenotypes and molecular pathogenesis of Marfan syndrome caused by cysteine mutations in different fibrillin-1 domains. These results strongly suggest that failure to form disulfide bonds and abnormal proteolysis of fibrillin-1 caused by cysteine mutations may be an important factor underlying the pathogenesis of diseases caused by fibrillin-1 mutations, such as Marfan syndrome.

## Introduction

Marfan syndrome (OMIM 154700) is a rare autosomal dominant disease with an incidence of 1-2/10,000, characterized by congenital abnormalities of the eye, bone, and cardiovascular development (Ho et al., 2005). The diagnosis of Marfan syndrome can be made according to the Ghent diagnostic standard (Loeys et al., 2010) and usually involves aortic dilatation and ectopia lentis, although skeletal features and *FBN1* genotyping can also be taken into account. The clinical manifestations of Marfan syndrome are highly heterogeneous between and within families. Marfan syndrome can be life-threatening due to cardiac abnormalities such as aortic aneurysm rupture. Therefore, it is very important to diagnose the disease as soon as possible and carry out corresponding prevention and treatment.

Mutations in the human fibrillin-1 (*FBN1*) gene are the major cause of Marfan syndrome (Milewicz et al., 2021). *FBN1* contains 66 exons, is located on chromosome 15q21.1, and encodes fibrillin-1 with a relative molecular weight of 350 kDa. Fibrillin-1 is a glycoprotein that is a component of the extracellular matrix and is primarily involved in microfibril formation (Reyes-Hernández et al., 2016). Fibrillin-1 primarily consists of 47 epidermal growth factor-like (EGF-like) domains, two-hybrid (Hyb) domains, and seven transforming growth factor-β-binding protein-like (TB) domains that are interspersed with EGF domains. The pathogenic variants are usually located in the EGF-like domains (Zeyer and Reinhardt, 2015). These mutations, which generally affect the cysteine residues or calcium-binding amino acids, lead to a change in single amino acid and prevent the formation of disulfide bonds or calcium binding, both of which are important for secondary structure. The site and type of *FBN1* mutations may be related to the clinical phenotype. Mutations in exons 1–15 often cause abnormal ocular phenotypes and cysteine replacement mutations in calcium-binding EGF-like (cbEGF-like) sequences usually cause cardiac problems (Child, 2017).

Ectopia lentis [EL; Online Mendelian Inheritance in Man (OMIM 129600)] is a genetic disease characterized by an abnormal lens suspensory ligament, which often occurs as a symptom of Marfan syndrome or as isolated ectopia lentis. Fibrillin-1 is the main component of the suspensory ligament of the lens and plays an important role in maintaining the morphological integrity and normal function of the connective tissue (Adès et al., 2010). In this study, using whole-exome sequencing we detected two *FBN1* mutations in two Chinese families with Marfan syndrome including lens dislocation. Using bioinformatics and recombinant peptide analyses of these two mutations, we investigated the different effects of these cysteine mutations in the non-cbEGF-like and cbEGF-like domains of fibrillin-1 on protein structure and stability, proteolytic capacity, and the possible pathogenesis of Marfan syndrome with ectopia lentis.

## Materials and methods

### Patients and clinical data

Two probands from Guangdong Province of China attended Shenzhen Eye Hospital because of unclear vision since childhood. Four patients (III: 3, III: 5, III: 7, and IV: 2) and four unaffected members [III: 2, III: 4 (the father of the proband), IV: 1, and IV: 3] of family 1 and one patient (III: 1) and the mother (II: 1) of the proband of family 2 were recruited for this study. After obtaining informed consent from all the participants and/or their legal guardians, the participants underwent complete physical and ophthalmic examinations. This study was approved by the Ethics Committee on Clinical Investigations of the Shenzhen Eye Hospital (no. 2021110402-01).

### Genomic DNA preparation and whole-exome sequencing

Approximately 5 ml peripheral blood samples were collected in EDTA tubes and genomic DNA was extracted from each sample using the MagPure Buffy Coat DNA Midi KF Kit (Qiagen, United States) according to the manufacturer’s instructions. Quantified genomic DNA samples were randomly fragmented using an ultrasonic high-performance sample processing system (Covaris) and fragments of approximately 280–320bp were obtained. DNA fragment end repair was then carried out, and the hybrid library was prepared using linear amplification mediated PCR (LM-PCR). An appropriate amount of hybridization library was captured and enriched using an exon chip (KAPA Hyper Exome, ROCHE), followed by elution and post-capture amplification. The amplification products were subjected to single-strand separation and cyclization, and the cyclized library generated DNA NanoBalls (DNBs) through rolling circle amplification (RCA). After quality control using the Agilent 2100 Bioanalyzer and ABI StepOne, high-throughput sequencing was performed on the MGISEQ-2000 platform (BGI, Guangdong, China). Raw reads generated by the sequencer were first screened and low-quality reads were discarded. Detailed data analysis and candidate gene mutation filtering criteria have been previously described (Al-Muhanna et al., 2019).

### Sanger sequencing

After the candidate gene mutations were identified, Sanger sequencing was further performed to validate the results. Primers were designed and synthesized upstream and downstream of the pathogenic gene mutation sites. PCR amplification was performed using SYBR Premix Ex Taq according to the manufacturer’s instructions (Takara, Dalian, China). The PCR products were then purified using a DNA purification kit (Sangon Biotech Co., Ltd., Shanghai, China) and sequenced using ABI 3730 DNA Analyzers (Hudson, New Hampshire, United States). Sequencing results were aligned with the *FBN1* reference sequence (NM_000138.4).

### Mutant protein function prediction

Human fibrillin-1 protein sequences were aligned with those of other species to analyze the conservation of mutant residues using Geneious. SIFT (http://sift.jcvi.org/www/SIFT_enst_submit.html), PolyPhen-2 (http://genetics.bwh.harvard.edu/pph2/), and PROVEAN (http://provean.jcvi.org/) software were used to predict the effect of the mutant proteins.

### Structure analysis

The SWISS-MODEL online server (https://www.swissmodel.expasy.org) was used to simulate the homology structure of fibrillin-1 based on the human wild-type (WT) FBN1^113−287^ (Protein Data Bank database, family with ID: 5MS9), and the structures of WT and mutation models of FBN1^1238−1445^ were modeled by the Alphafold software (Varadi et al., 2022). Three-dimensional (3D) models of the WT and mutant proteins were visualized using the Pymol software (v2.1).

Fibrillin-1 protein stability was evaluated using the web-based DUET tool (http://biosig.unimelb.edu.au/duet/stability) (Pires et al., 2014).

### Protein expression and purification

The human FBN1^113−287^ (FBN1^E2cbEGF1^), FBN1^113−287+C160Y^ (FBN1^E2cbEGF1+C160Y^) fragment, latent transforming growth factor beta-binding protein 1 (LTBP1)^1141-1395^ (LTBP1^cbEGF14cbEGF15^), FBN1^1238−1445^ (FBN1^cbEGF20-24^), and FBN1^1238−1445+C1350Y^ (FBN1^cbEGF20-24+C1350Y^) fragments were synthesized by NovoPro Bioscience Inc. (Shanghai, China). The corresponding cDNA fragments were ligated into a pCOLD II vector (NovoPro Bioscience Inc., Shanghai, China). The WT and mutant *FBN1* plasmids were transformed into *Escherichia coli* BL21 (DE3) cells. Protein expression was induced using 0.5 mM IPTG at 37°C for 4 h. The bacterial pellet was resuspended in 20 ml lysis buffer (20 mM Tris-HCl containing 1 mM PMSF and bacteria protease inhibitor cocktail, pH 8.0), the cell lysate was sonicated, and centrifuged at 4°C, 10000 rpm for 20 min, and the pellet was collected. The inclusion bodies were washed thrice with inclusion body washing solution (20 mM Tris, 1 mM EDTA, 2 M urea, 1 M NaCl, and 1% Triton X-100, pH 8.0). The inclusion bodies were collected and dissolved in 20 mM Tris, 5 mM DTT, 8 M urea (pH 8.0) and kept overnight at 4°C, followed by centrifugation at 10,000 rpm for 15 min at room temperature. To the obtained solution, 20 mM Tris-HCl, 0.15 M NaCl was added dropwise, gradually diluted in multiples, and slowly stirred, and the protein solution was placed into a dialysis bag and dialyzed overnight in a solution of 20 mM Tris-HCl, 0.15 M NaCl, pH 8.0. To gradually dilute the above solution and prepare multiple dilution gradients, 20 mM Tris-HCl, 0.15 M NaCl, pH 8.0 buffer was used, and the protein solution was placed into the dialysis bag for dialysis overnight. Using a low-pressure chromatography system, the supernatant solution was loaded onto a Ni-IDA-Sepharose Cl-6B affinity chromatography column pre-equilibrated with Ni-IDA-binding buffer at a flow rate of 0.5 ml/min. The target protein was eluted with Ni IDA elution buffer (20 mM Tris-HCl, 250 mM imidazole, 0.15 M NaCl, pH 8.0) at a flow rate of 1 ml/min, the effluent was collected and added to the dialysis bag, and PBS was used for dialysis overnight. SDS-PAGE analysis (12%) was performed, and Coomassie brilliant blue staining was used for band color development.

### Affinity determination

The binding between the fibrillin-1 and latent transforming growth factor beta-binding protein 1 (LTBP1) fragments was analyzed in real-time using the surface plasmon resonance (SPR) ProteOn system. The running buffer consisted of 10 mM HEPES, 150 mM NaCl, and 2 mM CaCl_2_, at pH 7.4, supplemented with 0.005% (v/v) Tween-20, and the running buffer reagent bottle was connected to Biacore T200. Running buffer was used to dialyze the analyte overnight, and then to dilute the analyte to form a concentration gradient. Different concentrations of analytes and 50 mM HCl regeneration solution were loaded into Eppendorf tubes, placed into the Biacore T200 sample compartment, and affinity determination was conducted.

### Protease degradation experiments

Four fibrillin-1 fragments were used for the protease degradation experiments. Protease was added according to a mass ratio of trypsin or chymotrypsin (Promega, Madison, WI) to the substrate of 1:50, shaken quickly, mixed well, and incubated in a water bath at 37 C, and timing was initiated. Samples were obtained at 0, 10, 30, and 60 min. Loading buffer (5X) was immediately added, and the samples were heated to 95°C for 5 min. The hydrolyzed protein samples were then subjected to SDS gel electrophoresis (15% acrylamide). Finally, the gels were stained with Coomassie brilliant blue.

### Liquid chromatography-tandem mass spectrometry (LC-MS/MS) and data analysis

50 mmol/L NH_4_HCO_3_ solution was added to 10 μg protein solution to a total volume of 100 μl. Then, iodoacetamide solution was added to a final concentration of 50 mmol/L, and the mixture reacted in the dark for 40 min. Trypsin or chymotrypsin was added at a mass ratio of enzyme to the substrate of 1:100 or 1:40, respectively, and the samples were digested for 16 h at 37°C. Following trypsin digestion, 15 μg peptides were desalted using a self-packing desalting column and then dried under a vacuum at 45°C. The LC-MS experiments were conducted using a Q Exactive™ Hybrid Quadrupole-Orbitrap™ mass spectrometer coupled with an Ultimate 3000 nano-liquid chromatograph (Thermo Fisher Scientific, UK). The peptides were dissolved in 0.1% formic acid and 2% acetonitrile and loaded onto a pre-column (Acclaim PepMap RPLC C18, 300 μm × 5 mm, 5 μm packing material, 100 Å). The trapping column was then set in line with an analytical column (Acclaim PepMap RPLC C18, 150 μm × 150 mm, 1.9 μm packing material, 100 Å). The mobile phases for LC separation were as follows: linear gradient of 95% A (0.1% formic acid in water), 5% B (80% acetonitrile, 0.08% formic acid), and 60% A: 40% B over 50 min at a flow rate of 300 nl/min. The mass spectrometer was operated in Data-Dependent Acquisition (DDA) mode to acquire a full MS scan with m/z 300–1,800 acquired at a mass resolution of 70,000.

The LC-MS/MS raw data were analyzed using MaxQuant for peptide identification against the reference proteome set of *Homo sapiens* from Uniprot (v1.6.2.10) (Costanzo et al., 2022). The peptide and fragment mass tolerance were set to 20 ppm, trypsin or chymotrypsin was selected as the proteolytic enzyme, and the maximum number of missed cleavages was two or three, respectively. Carbamidomethylation (Cys) was set as a fixed modification, while oxidation (Met) and N-terminal acetylation were the variable modifications. The peptide false-discovery rate was set to 1%.

## Results

**FIGURE 1:**
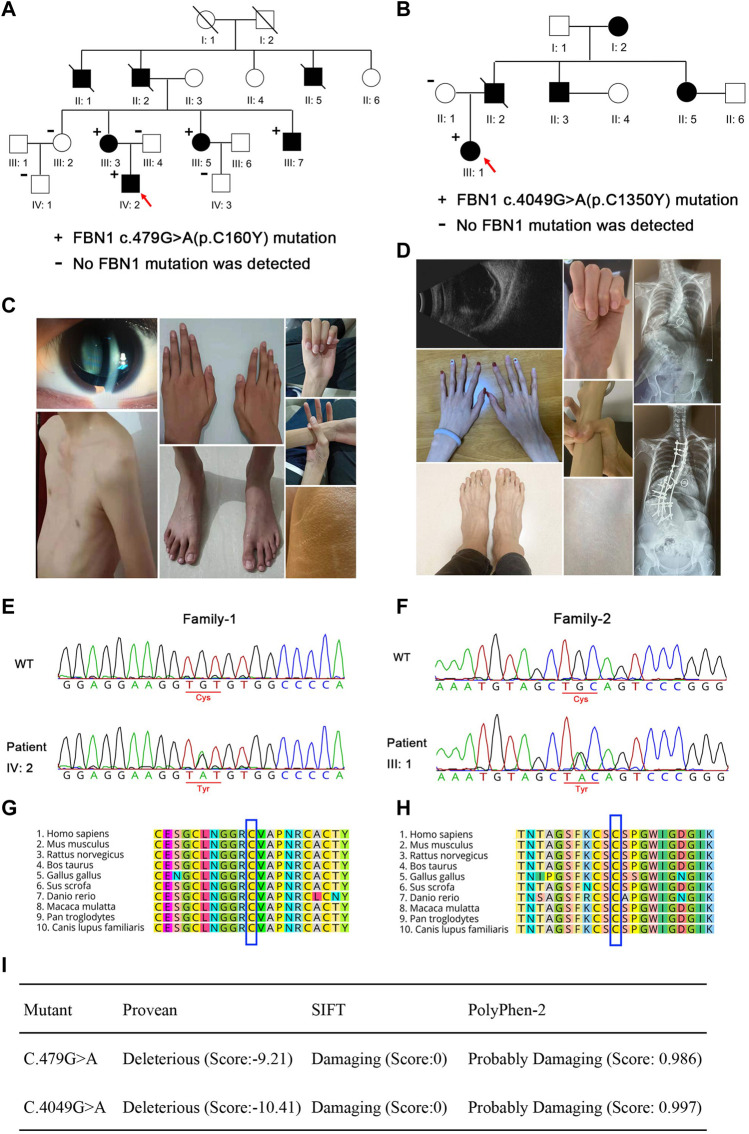
Pedigree and clinical characteristics of the two families. **(A,B)** Pedigree and clinical characteristics of family 1. **(C,D)** Pedigree and clinical characteristics of family 2. +, represents a heterozygous *FBN1* mutation. - represents the unaffected members who were tested for the variants. The filled squares (male) and circles (female) indicate the individual had ectopia lentis and minor signs of Marfan syndrome. Red arrows indicate the probands. Representative sequencing results show the FBN1 c.479G>A **(E)** and c.4049G>A **(F)** mutations in the family members with Marfan syndrome and the corresponding sequence in the unaffected family members. Conservation analysis in multiple species for p. C160Y **(G)** and p. C1350Y **(H)**. **(I)** Pathogenicity prediction of FBN1 mutations.

Through detailed examination of the ocular, skeletal, and cardiovascular systems of the participants in the two families, we found that both families had one individual with aortic aneurysm and ectopia lentis (III: 7 in family 1 and II: 3 in family 2), meeting the criteria for Marfan syndrome independent of family history, while other affected members had ectopia lentis and minor signs, meeting the criteria in the presence of family history (Loeys et al., 2010) (Figures 1A,C; Table 1). The proband in family 2 had undergone mitral valve replacement and her father had died due to a ruptured aortic aneurysm. Ocular examination showed that the proband in family 1 had dislocation of the right lens, and the left eye had undergone lens extraction and intraocular lens implantation 9 years ago at our hospital; the proband in family 2 had also undergone binocular intraocular lens implantation due to lens dislocation and suffered from binocular retinal detachment (Figures 1B,D). Additionally, none of the unaffected family members examined had any signs or symptoms of Marfan syndrome and none had a systemic score of 7 or greater.

**TABLE 1:** The clinical features of patients with Marfan syndrome identified in the present study.

Patient ID			Family 1				Family 2		
			III: 3	III: 5	III: 7	IV: 2	II: 3	II: 5	III: 1
Age (years)			39	37	34	12	45	40	28
Sex			Female	Female	Male	Male	Male	Female	Female
Height (cm)			172	176	199	170	189	172	174
Weight (kg)			57.5	59	72	40	63	51	45
Systemic features		Wrist and thumb sign	-	+	+	+	+	-	+
		Pectus excavatum	-	-	-	+	+	-	+
		plain pes planus	-	-	-	-	-	-	+
		Pneumothorax	-	-	-	-	-	-	-
		Dural ectasia	/	/	/	/	/	/	+
		Protrusio acetabuli	-	-	-	-	-	-	-
		Reduced US/LS AND increased arm/height AND no severe scoliosis	+	+	+	+	+	+	-
		Scoliosis or thoracolumbar kyphosis	+	-	-	-	+	+	+
		Reduced elbow extension	-	-	-	-	-	-	-
		Facial features	-	-	-	-	-	-	-
		Skin striae	+	+	+	+	+	+	+
		Myopia > 3 diopters	+	+	+	+	+	+	+
		Mitral valve prolapse	+	-	+	-	+	+	+
		**Systemic Score**	**5**	**6**	**7**	**7**	**9**	**5**	**11**
Others	Ectopia lentis		+	+	+	+	+	+	+
	Retinal detachment		-	-	-	-	+	-	+
	Aortic aneurysm		-	-	+	-	+	-	-

+, Present; -, absent; /, unknown.

Whole-exome sequencing and Sanger sequencing of the patients and their family members revealed that the patients in family 1 (III: 3, III: 5, III: 7, and IV: 2) carried the c.479G>A heterozygous mutation, while the proband in family 2 (III: 1) carried the c.4049G>A heterozygous mutation. These gene mutations were not found in the normal members of the two families (III: 2, III: 4, IV: 1, and IV: 3 in family 1, and II: 1 in family 2) (Figures 1E,F). Mutations in families 1 and 2 resulted in cysteine to tyrosine substitution at positions 160 and 1350 of fibrillin-1, respectively. Protein homologous sequence analysis revealed that the two mutation sites were highly conserved in the fibrillin-1 proteins of different species (Figures 1G,H). SIFT and PolyPhen-2 predicted that the mutation site may cause the destruction of the structure and function of fibrillin-1 (Figure 1I).

**FIGURE 2:**
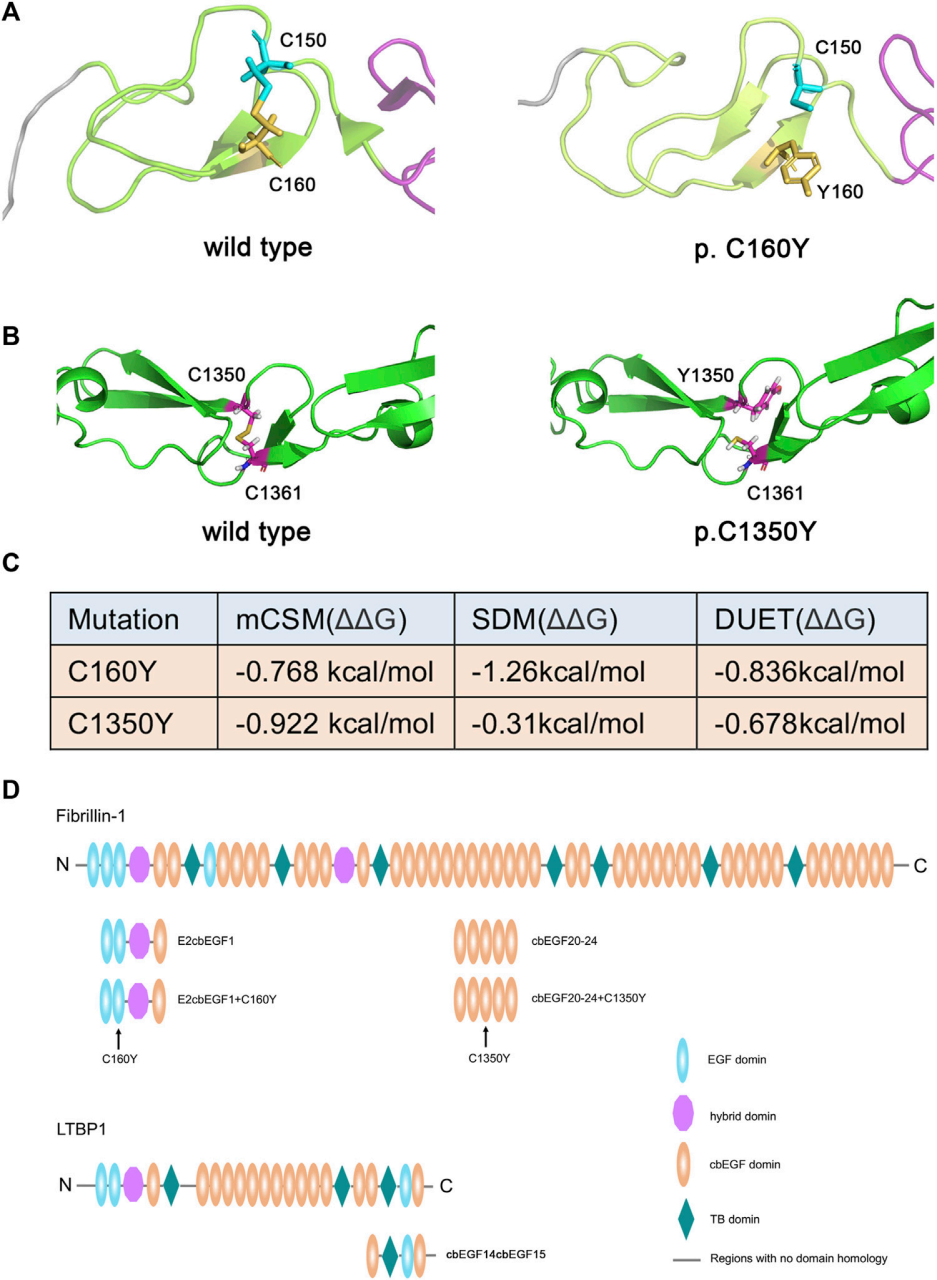
Effects of variants on the structure and stability of the fibrillin-1 protein. **(A)** Three-dimensional structural map of the EGF2 domain to cbEGF1 domain of fibrillin-1 without (left) or with the C160Y mutation (right). **(B)** Three-dimensional structural map of the cbEGF20 domain to cbEGF24 domain of FBN1 without (left) or with the C1350Y mutation (right). **(C)** Stability analysis of the wild-type and mutant fibrillin-1 proteins. **(D)** Schematic drawing of the recombinant polypeptides. The position of the fibrillin-1 C160Y and C1350Y mutations are indicated by arrows.

Bioinformatic analysis predicted that residues 150 and 160, as well as residues 1350 and 1361 of fibrillin-1, can form disulfide bonds, while the C160Y and C1350Y mutations would lead to the inability to form these disulfide bonds, thus affecting the stability of the protein structure. As shown in Figures 2A,B, the C160Y mutation resulted in significant changes in the fibrillin-1 structure, while the C1350Y mutation resulted in slight changes in the fibrillin-1 structure. The effects of the two *FBN1* mutations on fibrillin-1 protein stability were predicted using the DUET software, and the results showed that these mutations could cause fibrillin-1 protein destabilization (Figure 2C).

**FIGURE 3:**
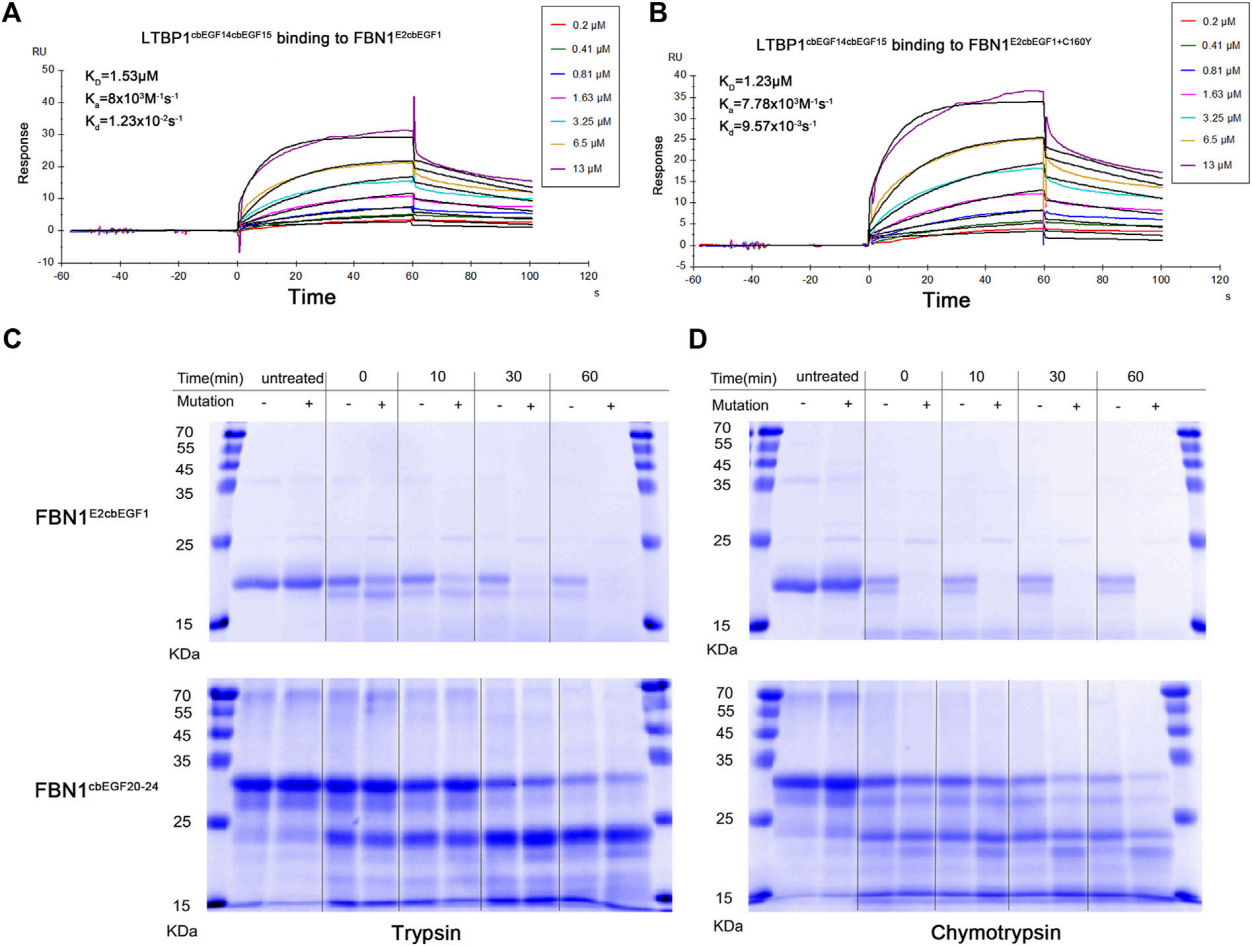
Affinity and Proteolytic degradation of the wild-type and mutant recombinant fibrillin-1 polypeptides. Affinity analysis of wild-type fibrillin-1 polypeptides (FBN1^E2cbEGF1^) **(A)** or mutant recombinant fibrillin-1 polypeptides (FBN1^E2cbEGF1+C160Y^) **(B)** and LTBP1 polypeptides (LTBP1^cbEGF14cbEGF15^). Wild-type (-) or mutant (+) recombinant FBN1 ^E2cbEGF1^ polypeptides **(C)** and FBN1^cbEGF20-24^ polypeptides **(D)** were incubated with trypsin (left) or chymotrypsin (right) for untreated, 0, 10, 30, and 60 min, and analyzed using SDS gel electrophoresis and Coomassie brilliant blue staining. Reduced marker proteins are indicated on the left in kDa.

It has been reported that the Hyb1 and EGF2/EGF3 domains of fibrillin-1 are involved in the binding of LTBP1 protein and that the C160Y mutation in the EGF3 domain may affect this interaction. Therefore, we prepared recombinant fibrillin-1 and LTBP1 proteins. Because of the large size of fibrillin-1 and LTBP1, the full-length recombinant proteins are prone to protein folding errors. Therefore, we prepared fibrillin peptides containing the EGF2, EGF3, Hyb1, and cbEGF1 domains, and LTBP1 peptides containing the cbEGF14, TB3, EGF3, and cbEGF15 domains (Figure 2D). The affinity of LTBP1^cbEGF14cbEGF15^ for FBN1^E2cbEGF1^ and FBN1^E2cbEGF1+C160Y^ was tested using SPR technology. The results showed that the dissociation constant (K_D_) of FBN1E2cbEGF1 and LTBP1 was 1.53 μM (Figure 3A), and that of FBN1^E2cbEGF1+C160Y^ and LTBP1 was 1.23 μM (Figure 3B); therefore, the C160Y mutation did not affect the binding affinity of fibrillin-1 and LTBP1.

Moreover, we analyzed the effect of cysteine mutations on proteolytic sensitivity following trypsin and chymotrypsin treatments. For the FBN1^E2cbEGF1^ and FBN1^E2cbEGF1+C160Y^ polypeptides, after heat denaturation, the degradation rate of the mutant peptide was significantly higher than that of the WT peptide. Both were degraded into small fragments with increasing digestion time. After 60 min of trypsin digestion or 30 min of chymotrypsin digestion, the mutant polypeptide was completely hydrolyzed, whereas the WT polypeptide was only partially hydrolyzed (Figure 3C). For the FBN1^cbEGF20-24^ and FBN1^cbEGF20-24+C1350Y^ polypeptides, the thermostability and hydrolysis sensitivity of the two peptides did not significantly change after trypsin digestion, but the thermostability of the mutant polypeptides decreased and hydrolysis sensitivity increased after chymotrypsin digestion (Figure 3D).

Using LC-MS/MS detection, we further analyzed the restriction sites in the WT and mutant polypeptides. In theory, the cleavage sites of trypsin were the same in the WT and mutant peptides. However, we only found the FBN1^C160-R180^ peptide in the degradation products of the WT peptides but not in those of the mutant peptides (Table 2). As shown in Table 2, the cleavage site of the FBN1^C136-R170^ peptide fragment was changed by treating the mutant polypeptide with chymotrypsin, and the cleavage site was changed from position 140 to position 160. Similarly, the C1350Y mutation created a new cleavage site in the FBN1^K1347-W1354^ peptide, which is consistent with the increased sensitivity of peptides to proteolysis under the action of chymotrypsin.

**TABLE 2:** Summary of product sequences obtained after protease digestion of the FBN1^E2cbEGF1^ peptides, FBN1^E2cbEGF1+C160Y^ peptides, FBN1^cbEGF20-24^ peptides, and FBN1 ^cbEGF20-24+C1350Y^ peptides.

Peptide	Protease	Peptide	Fragment
FBN1^E2cbEGF1^/ FBN1^E2cbEGF1+C160Y^	Trypsin	S^115^IQHCNIR^122^	Both
		G^139^YIGTHCGQPVCESGCLNGGR^159^	Both
		C^160^VAPNR^165^	FBN1^E2cbEGF1^
		C^160^VAPNRCACTYGFTGPQCER^179^	FBN1^E2cbEGF1^
		Y^160^VAPNR^165^	FBN1^E2cbEGF1+C160Y^
		C^166^ACTYGFTGPQCER^179^	Both
		T^183^GPCFTVISNQMCQGQLSGIVCTK^206^	Both
		T^207^LCCATVGR^215^	Both
		R^233^GFIPNIR^240^	Both
		G^234^FIPNIR^240^	Both
		L^279^NEVSQK^285^	Both
	Chymotrypsin	C^136^QKGY^140^	FBN1^E2cbEGF1^
		C^136^QKGYIGTHCGQPVCESGCLNGGRY^160^	FBN1^E2cbEGF1+C160Y^
		I^141^GTHCGQPVCESGCLNGGRCVAPNRCACTY^170^	FBN1^E2cbEGF1^
		V^161^APNRCACTY^170^	FBN1^E2cbEGF1+C160Y^
		G^171^FTGPQCERDY^181^	Both
		G^171^FTGPQCERDYRTGPCF^187^	Both
		R^182^TGPCF^187^	Both
		S^200^GIVCTKTL^208^	Both
		C^209^CATVGRAW^217^	Both
		A^211^TVGRAW^217^	Both
		I^236^PNIRTGACQDVDECQAIPGL^256^	Both
		C^257^QGGNCINTVGSF^269^	Both
		C^257^QGGNCINTVGSFECKCPAGHKLNEVSQKCEL^288^	FBN1^E2cbEGF1^
		E^270^CKCPAGHKL^279^	Both
FBN1^cbEGF20-24^/ FBN1 ^cbEGF20-24+C1350Y^	Trypsin	T^1255^NIPGEYR^1262^	Both
		C^1263^LCYDGFMASEDMK^1276^	Both
		T^1277^CVDVNECDLNPNICLSGTCENTK^1300^	Both
		G^1301^SFICHCDMGYSGK^1314^	Both
		T^1318^GCTDINECEIGAHNCGK^1335^	Both
		H^1336^AVCTNTAGSFK^1347^	Both
		C^1348^SCSPGWIGDGIK^1360^	FBN1^cbEGF20-24^
		C^1348^SYSPGWIGDGIK^1360^	FBN1^cbEGF20-24+C1350Y^
		N^1382^TMGSYR^1388^	Both
		N^1417^GQCLNAPGGYR^1428^	Both
		C^1429^ECDMGFVPSADGK^1442^	Both
	Chymotrypsin	M^1270^ASEDMKTCVDVNECDL^1286^	Both
		S^1293^GTCENTKGSF^1303^	Both
		I^1304^CHCDMGY^1311^	Both
		H^1336^AVCTNTAGSF^1346^	Both
		K^1347^CSCSPGW^1354^	FBN1^cbEGF20-24^
		S^1351^PGW^1354^	FBN1^cbEGF20-24+C1350Y^
		I^1355^GDGIKCTDL^1364^	Both
		A^1378^DCKNTMGSY^1387^	Both
		R^1388^CLCKEGY^1395^	Both
		T^1396^GDGF^1400^	Both
		N^1413^LCGNGQCLNAPGGY^1427^	Both
		R^1428^CECDMGF^1435^	Both

FBN1, fibrillin-1; cbEGF, calcium-binding epidermal growth factor.

## Discussion

Marfan syndrome is a connective tissue disease mainly caused by pathogenic variants of *FBN1*, which encodes the extracellular matrix protein fibrillin-1. Fibrillin-1 is the main component of the extracellular microfibril skeleton and is widely expressed in elastic tissues, such as the skin, cardiovascular system, tendon, and non-elastic tissues, such as the lens suspensory ligament (Kielty et al., 2002). The correlation between the *FBN1* genotype and the Marfan syndrome phenotype is complex, involving different mutation types (Child, 2017). Based on their effect on protein structure, *FBN1* mutations can be divided into two types: missense mutations, and protein-truncating mutations (Xu et al., 2020). Mutations in *FBN1* may destroy the integrity of the fiber structure and extracellular matrix, cause connective tissue abnormalities, and lead to the accumulation of the fibrillin-1 protein in multiple organs in the whole body (Sengle and Sakai, 2015). In this study, two recurrent *FBN1* mutations, c.479G>A (p.C160Y) and c.4049G>A (p.C1350Y) were found in two Chinese families with Marfan syndrome with aortic aneurysm and ectopia lentis. The c.479G > A mutation was previously detected in a classic Marfan family, but detailed clinical data are lacking (Stheneur et al., 2009). In a previous study, a similar mutation, c.478T > C (p.C160R), was identified in a child with incomplete Marfan syndrome with ocular system abnormalities (Comeglio et al., 2010). The c.4049G>A mutation was detected in a family with Marfan syndrome with aortic regurgitation and thoracic aortic aneurysm, but it was not mentioned if it was accompanied by ocular abnormalities, such as lens dislocation and retinal detachment (Stheneur et al., 2009; Fang et al., 2017).

Fibrillin-1 is widely distributed in ocular tissue, including the lens suspensory ligament, lens capsule, and connective tissue (Wheatley et al., 1995). The fibrillin-1 positive expression has also been observed in the corneal epithelium and endothelial cells, sclera, and choroid (Wheatley et al., 1995). Mutation of fibrillin-1 leads to abnormal development of the suspensory ligament of the lens, resulting in lens dislocation, which is a common ocular phenotype in patients with Marfan syndrome (Chandra et al., 2014; Sridhar and Chang, 2017). Compared to the normal control group, the number of ribbon fibers in the ectopic lens decreased and became thinner, inelastic, and easy to break (Kielty et al., 1995; Mir et al., 1998). Ectopia lentis causes traction on the base of the vitreous body, causing small tears or holes around the retina, which can cause severe retinal detachment (Remulla and Tolentino, 2001; Chandra et al., 2014). In our study, patients carrying C160Y or C1350Y mutations had ectopia lentis, while the patient carrying the C1350Y mutation also had retinal detachment. The two mutations are located in the EGF3 and cbEGF22 domains, respectively, which is consistent with the finding that individuals with cysteine replacement or splice-site mutations are more likely to develop ocular system abnormalities (Schrijver et al., 1999). A recent study found that patients with cysteine substitutions or calcium-binding mutations in fibrillin-1 cbEGF-like domains had more severe ocular involvement (Zhang et al., 2021). Moreover, different types or different domains of *FBN1* mutations are associated with different ocular phenotypes (Chen et al., 2021). In addition, eye abnormalities such as high myopia, long axis, and glaucoma have also been reported in Marfan syndrome (Kielty et al., 1995; Nazarali et al., 2017; Singh et al., 2021).

Like many other extracellular glycoproteins, fibrillin-1 consists largely of a single module repeated multiple times (Ramirez and Pereira, 1999). Each EGF-like domain of fibrillin-1 forms three disulfide bonds between the six cysteine residues in each domain in a C1–C3, C2–C4, and C5–C6 pattern (Downing et al., 1996). The C160Y mutation leads to the deletion of the third cysteine in the EGF3 domain, resulting in the failure to form the C1–C3 disulfide bond, while the C1350Y mutation leads to the deletion of the fifth cysteine in the cbEGF-like domain, resulting in failure to form the C5–C6 disulfide bond (Figure 3). Substitution or addition of cysteine residues may result in free reactive cysteine residues or induce disulfide bond shuffling and misfolding, altering the protein structure and causing proteins to be more easily hydrolyzed *in vitro* (Zeyer and Reinhardt, 2015). Based on these assumptions, we generated recombinant proteins containing C160Y and C1350Y mutations to analyze their effect on fibrillin-1 function. Because the recombinant protein of full-length fibrillin-1 is prone to aggregation, leading to structural errors, we only prepared recombinant peptides to analyze the function of the mutant protein (Vollbrandt et al., 2004). Although the effects on protein function of cysteine mutations in the cbEGF-like domain have been previously investigated, the effects of cysteine mutations in the non-cbEGF-like domain have not.

Since the C160Y mutation is located in the EGF3 domain of fibrillin-1, and it has been reported that the EGF2-EGF3 regions of fibrillin-1 interact with the LTBP1 (Robertson et al., 2017), we tested the affinity of FBN1^E2cbEGF1^ and FBN1^E2cbEGF1+C160Y^ for LTBP1^cbEGF14cbEGF15^ to determine if abnormal affinity was involved in the pathogenic process. The results showed that their affinities for LTBP1 were not significantly different, indicating that the disease caused by the fibrillin-1 C160Y mutation was not due to an altered affinity for LTBP1.

Both fibrillin-1 molecules and fibrillin-rich microfibrils are susceptible to serine protease cleavage (e.g., cleavage by chymotrypsin and trypsin). Increased proteolytic susceptibility may lead to the degradation of fibrillin-1 monomers, leading to reduced fibrillin-1 protein incorporation into microfibrils, or to fibrillin-1 protein degradation in assembled microfibrils, resulting in microfibril dysfunction (Vollbrandt et al., 2004). In our study, we found that compared with the FBN1^E2cbEGF1^ polypeptide, the FBN1^E2cbEGF1+C160Y^ polypeptide had decreased thermal stability and was more sensitive to proteolytic degradation by trypsin or chymotrypsin. However, for the FBN1^cbEGF20-24^ and FBN1^cbEGF20-24+C1350Y^ polypeptides, there was no significant difference in the proteolytic sensitivity under trypsin treatment, but the protein degradation of the mutant peptide was significantly increased after chymotrypsin treatment for 30 min. This is related to the different cleavage sites of different enzymes. Mutation of cysteine to tyrosine in the non-cbEGF-like and cbEGF-like domains both changed the recognition site of chymotrypsin, making the protein more susceptible to hydrolysis, which was confirmed using LC-MS/MS analysis. Compared to the cbEGF-like domain, the non-cbEGF-like domain lacks an aspartate at position 1 of the domain, and most mutations that cause Marfan syndrome occur in the cbEGF-like domain (Piersall et al., 1994). Our results showed that the peptide with the mutation in the non-cbEGF-like domain was more easily hydrolyzed than that with the mutation in the cbEGF-like domain, which may be due to the role of calcium-binding in stabilizing the fibrin structure, or due to the instability of the N-terminal domain resulting in poor protein thermal and hydrolytic stability (Kielty et al., 1994; Reinhardt et al., 1997; Smallridge et al., 1999). Since proteolytic sensitivity depends on the recognition site of the protease, and the cleavage sites of different proteases are not the same, the application of more types of proteases may be of great significance to comprehensively define the proteolytic sensitivity of mutants.

## Conclusion

In this study, we report the clinical phenotypes and molecular features of two patients with Marfan syndrome with recurrent fibrillin-1 cysteine mutations in the EGF and cbEGF domains. Both mutations lead to the inability to form disulfide bonds in fibrillin-1, resulting in changes in thermostability and trypsin and chymotrypsin sensitivity; these changes were more significant in the protein harboring the cysteine mutation in the EGF domain. Therefore, cysteine mutations in fibrillin-1 play an important role in the pathogenesis of Marfan syndrome. However, the pathogenic mechanisms underlying the effects of cysteine mutations in the EGF and cbEGF domains on protein function may not be completely consistent.

## Data Availability

The datasets for this article are not publicly available due to concerns regarding participant/patient anonymity. Requests to access the datasets should be directed to the corresponding authors.
